# Oral health literacy and its related factors among community-dwelling older adults: a mixed-methods study

**DOI:** 10.3389/fpubh.2026.1833803

**Published:** 2026-07-03

**Authors:** Yiqing Liang, Jingjing Wang, Peng Gao, Huiling Xu, Shuhua Wang, Man Feng, Songmei Cao

**Affiliations:** 1 Affiliated Hospital of Jiangsu University, Zhenjiang, China; 2 Affiliated Stomatological Hospital of Nanjing Medical University, Nanjing, China; 3 Jiangsu College of Nursing, Huaian, China

**Keywords:** older adults, oral health, oral health literacy, mixed methods, related factors

## Abstract

**Objective:**

This study examined oral health literacy (OHL) and its related factors among community-dwelling older adults.

**Methods:**

A mixed-methods design was employed. Community-dwelling older adults from Zhenjiang, China, were recruited between September and November 2021. Validated scales were used to assess oral health knowledge, oral health beliefs, OHL, self-efficacy, and oral health behaviors. Semi-structured interviews were conducted with 20 participants. Data integration adopted a synchronous two-way approach.

**Results:**

A total of 445 seniors completed the quantitative survey. Factors associated with OHL included dental cleaning, oral health behaviors, oral health knowledge and beliefs, educational attainment (high school or university level), self-efficacy, and difficulties in communicating with dentists. Four themes were identified from the qualitative study. Data integration revealed that oral health demands, knowledge, behaviors, beliefs, educational level, self-efficacy, and patient-dentist relationships were associated with OHL levels.

**Conclusion:**

Community-dwelling older adults exhibited moderate OHL associated with multiple factors. Future interventions should target oral health knowledge, beliefs, behaviors, and self-efficacy to enhance OHL in this population.

## 1 Introduction

Oral health literacy (OHL) refers to an individual’s ability to acquire, process, and understand basic health information and services necessary for making appropriate oral health decisions (1). Previous studies have shown that low OHL negatively affects oral health (2). Furthermore, OHL is also essential for reducing oral health disparities and promoting oral health (3–5). People with low oral health literacy are more likely to experience severe periodontitis, higher plaque scores, and severe tooth loss, all of which contribute to poor oral health (6, 7). Oral health not only affects physiological functions such as chewing and speech, but is also closely linked to systemic diseases, including cardiovascular and cerebrovascular diseases, diabetes mellitus, and Alzheimer’s disease (8–10). Poorer oral health imposes a greater financial burden. The cost of oral diseases worldwide reached $544 billion in 2015, ranking third behind diabetes and cardiovascular diseases (11). Therefore, improving OHL is considered a priority for promoting good oral health and preventing oral diseases (1).

Global population ageing has accelerated in recent decades. By 2050, the number of older persons globally is estimated to reach 1.5 billion (16% of the total population) (12), posing increasing challenges for public health policy. China, as the world’s largest developing country, had 296.97 million people aged 60 and over in 2023, accounting for 21.1% of the total population (13), and this number is projected to exceed 400 million by 2033 (14). Oral health is a critical aspect of healthy ageing, as poor oral health is a risk factor for oral pain, impaired mastication, systemic infections, malnutrition, and systemic diseases (15). Currently, 3.5 billion people worldwide suffer from dental caries, periodontal disease, tooth loss, and other oral conditions, among whom older adults bear a disproportionately high burden (16). According to the Fourth Chinese National Oral Health Epidemiologic Survey, 98.40% of the population aged 65–74 years had caries, 82.60% had bleeding gums, 90.30% had dental calculus, and only 18.30% retained intact teeth (17).

Despite rapid population ageing and the increasing prevalence of oral diseases, OHL in older adults has received limited attention from researchers. Existing research on OHL among older adults has been predominantly quantitative, examining relationships between OHL and specific outcomes such as oral health-related quality of life (particularly gender differences), the number of remaining teeth, and socioeconomic status (18–20). Other studies have focused on assessment tools, such as specific OHL instruments (21), or have employed network modelling to explore the usability of the OHL questionnaire among older adults (22). Our previous research explored the association between OHL and oral health behaviors among community-dwelling older adults using the IMB model, laying a solid foundation for the present study (23).

However, the factors associated with OHL in older adults are complex and difficult to fully elucidate through quantitative research alone. Qualitative studies can complement and deepen the interpretation of quantitative findings.

Therefore, we conducted this convergent mixed-methods study to achieve a more comprehensive understanding of the OHL characteristics and their associated factors among older adults. The objectives of this study were: (1) to profile the level and characteristics of OHL among community-dwelling older adults; (2) to determine the factors associated with OHL among community-dwelling older adults.

## 2 Methods

### 2.1 Study design

**Figure 1:**
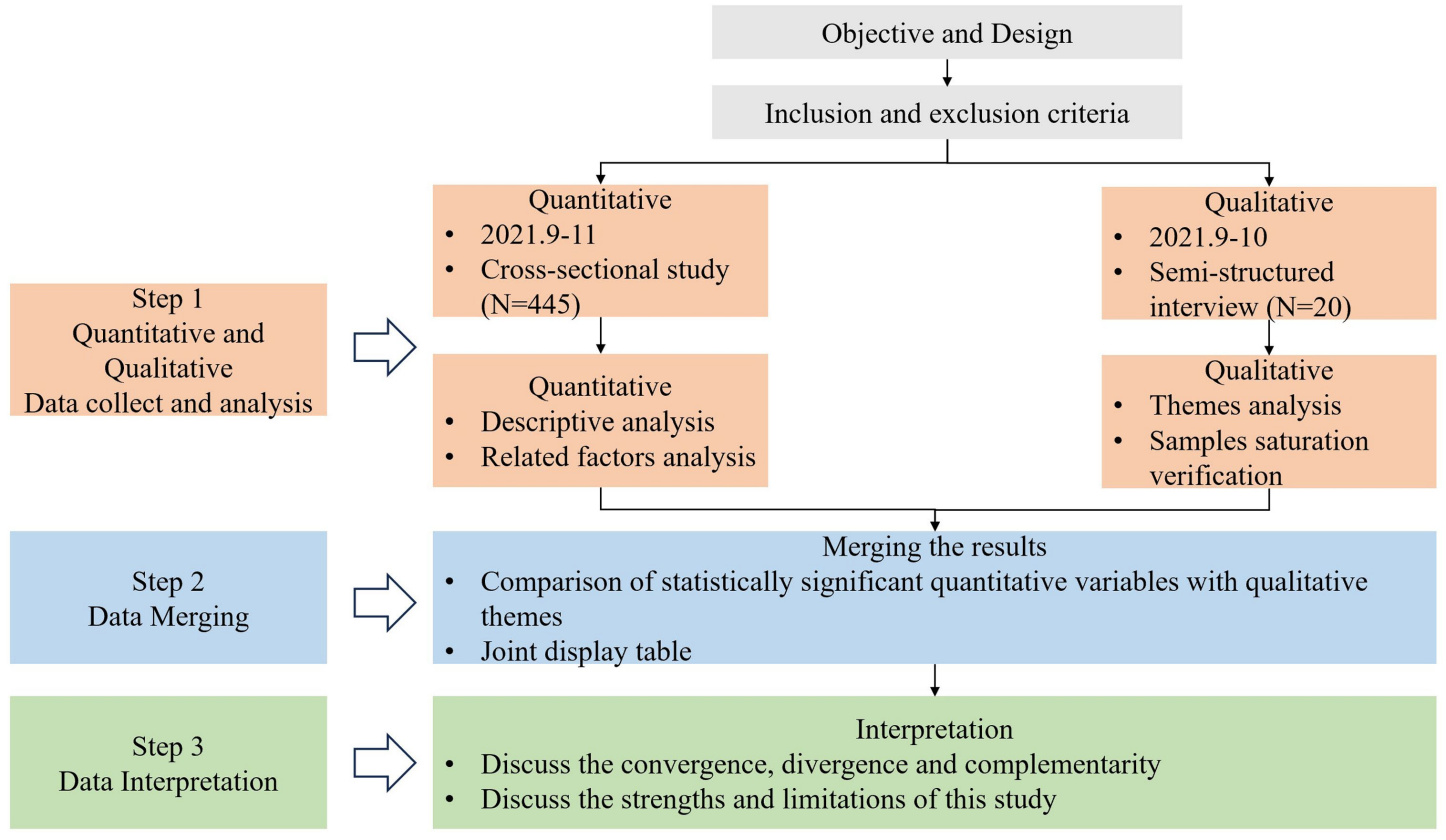
The flow diagram of study design.

This study adopted a convergent mixed-methods design (24). Quantitative and qualitative data were collected concurrently to comprehensively explore oral health literacy and its related factors among community-dwelling older adults. Quantitative data were used to identify key related factors, while qualitative data were used to explore barriers to accessing, understanding, and processing oral health information (see Figure 1). Variable selection was guided by our prior IMB-based findings (23). This study was reported in accordance with the Good Reporting of A Mixed-Methods Study (GRAMMS) checklist (25).

### 2.2 Study setting

Zhenjiang is located in southeastern Jiangsu Province, China, and serves as a pilot city for the reform of community-based senior care services nationwide. In 2022, it had 781,000 older people over the age of 60, accounting for 23.56% of the total population (26). Considering its economic level, size of older, and other relevant factors, the community senior service center in Jingkou District of Zhenjiang was chosen as the main study site.

### 2.3 Participants

#### 2.3.1 Quantitative research

Convenience sampling was used in the Zhenjiang Jingkou District Community Senior Service Center from September to November 2021. Participants were eligible if they met the following inclusion criteria: (a) age 60 years and older, (b) able to communicate and comprehend Mandarin, (c) able to perform activities of daily living independently, and (d) willing to participate in the survey. The exclusion criteria were: (a) a diagnosis of cognitive or psychiatric disorders; (b) had another serious physical disorder (e.g., advanced cancer, end-stage renal failure); and (c) inability to communicate effectively with the researcher. For multivariate analysis, the sample size was estimated to be 10–20 times the number of questionnaire variables (27). Therefore, a sample of approximately 290 participants was required. Finally, 445 questionnaires were distributed.

#### 2.3.2 Qualitative research

Data collection was implemented in the same community senior center in September–October 2021. To ensure maximum variation in oral health self-management practices and communication abilities, a purposive maximum variation sampling strategy was employed, considering age, education, tooth loss status, and other factors. Twenty participants ultimately took part in the interviews.

### 2.4 Data collection

In convergent mixed-method designs, both datasets are analyzed separately and then integrated (24).

#### 2.4.1 Quantitative research

The questionnaire consisted of four sections. (1) A self-designed socio-demographic questionnaire: it included age, gender, place of residence, occupation, monthly household income, residency status, smoking, and other information. (2) The Oral Health Literacy Adult Questionnaire(OHL-AQ): it was developed by Sistani et al. for community-dwelling adults (28). The total score ranges from 0 to 17, with 1 point for each correct answer. Total scores of 0 to 9 indicate inadequate OHL, 10 to 11 indicate moderate OHL, and 12 to 17 indicate high OHL. The Chinese version of this scale has a Cronbach’s *α* of 0.77 (29). (3) The Oral Health Knowledge, Belief, and Behavior Questionnaire for Community-Dwelling Older Adults: it was designed by Sheng Ye et al. (30). The scale includes 47 items and 3 dimensions. The oral health knowledge dimension has a maximum score of 19, and the oral health belief dimension has a maximum score of 17, with 1 point for each correct answer. The higher the score, the better the oral health knowledge and belief. Oral health behavior ranges from 1 (never) to 5 (always) for each item. Better scores signify good oral health behavior. The Cronbach’s *α* for the three dimensions were 0.827, 0.816, and 0.856, respectively. (4) the Self-efficacy Scale for Oral Health Behavior (SEOH): it was developed by Soutome et al. (31). The scale was translated into Chinese, and its psychometric properties were validated (32). The SEOH is a five-point Likert scale with 25 items classified into 4 categories. Higher overall scores indicate greater self-efficacy in oral health. The score ranges from 25 to 125. For the Chinese version of this scale, the Cronbach’s *α* is 0.93 (32). Quantitative data were collected through a face-to-face survey. The researchers were three uniformly trained postgraduate nursing students. They were asked to obtain participant consent prior to the survey and to inform all participants of the purpose and content of the study before the start of the survey. During the survey, a uniform presentation was provided to guide participants through the process of answering questions. Researchers assisted participants in completing the questionnaires to ensure standardization.

#### 2.4.2 Qualitative research

Qualitative data were collected through semi-structured individual interviews. The interviews focused on participants’ dilemmas in accessing and understanding oral health information, oral health self-management, and healthcare decision-making. Interview outlines were developed based on a literature review by two researchers working in geriatric care (Supplementary Materials 1). One researcher with extensive experience in geriatric care conducted all interviews independently. The interviews were conducted in a separate room at the community senior center, lasting 30–45 min. Before obtaining written consent, all participants were informed of the purpose, its significance, and the right to withdraw from the study at any time. Participants were also informed that the interviews were audio-recorded and transcribed. Reflective statements were written immediately after each interview and discussed weekly to improve the veracity of data and identify potential biases.

### 2.5 Data analysis

#### 2.5.1 Quantitative

All data were validated and checked by two researchers. Data analysis was performed using IBM SPSS v26.0. Descriptive analysis was conducted on the collected data according to variable types and distributions (mean, median, standard deviation, frequency, and proportion). The Mann–Whitney *U*-test and Kruskal–Wallis H test were used to assess demographic differences in OHL among participants. Spearman’s correlation analysis was used to assess the correlations among OHL, oral health knowledge, oral health beliefs, behaviors, and self-efficacy. As the residuals of the dependent variable (OHL) met the assumptions of linearity, independence, homoscedasticity, and normality, multiple linear regression was performed to explore factors associated with OHL (33). The total OHL score was used as the dependent variable, and all variables with statistically significant differences in univariate analyses were simultaneously entered into the model as independent variables. Categorical variables were dummy coded (Supplementary Materials 1). Multicollinearity was assessed using variance inflation factors (VIF < 10) to ensure model stability. Model fit was evaluated using *R*^2^ and adjusted *R*^2^. All tests were two-sided, with a significance level of 0.05.

#### 2.5.2 Qualitative

Data analysis and data collection were conducted simultaneously. NVivo 11.0 was used for transcription management. Data analysis employed inductive content analysis (34), comprising preparation, organization, and reporting phases. In the preparation phase, the third and fourth authors were responsible for transcription. The first and second authors conducted multiple in-depth reviews of each transcription and reflective statement. Any disagreements were resolved through discussion until consensus was achieved. In the organization phase, the first and second authors independently read the text line by line, writing annotations and headings to describe various aspects of the content. Headings were then collected to form a list of categories, which were subsequently abstracted into higher-level categories based on similarities or differences. After comparing and discussing the distinct categories identified by each author independently, four themes were established. During the reporting phase, narratives representing the themes were developed based on the data material, and all findings were systematically presented. No new information emerged after 16 interviews. However, four additional participants were recruited to ensure data saturation (35).

### 2.6 Data integration

We employed Creswell and Plano Clark’s data synthesis procedures for the convergent mixed-methods research design, utilizing joint display tables (24). Joint display tables, commonly used in mixed-methods designs, visually present both qualitative and quantitative findings alongside interpretive meta-inferences (36). Data integration adopted a simultaneous bidirectional approach (35). The bidirectional approach was applied after quantitative results and qualitative themes had emerged. Quantitative findings provided explanatory support for key qualitative findings to confirm, disconfirm, and extend established meta-inferences (24).

### 2.7 Rigour

In convergent mixed-methods research designs, threats to validity must be minimized. In the quantitative phase, all measurement instruments demonstrated high reliability. Researchers received standardized training before data collection. To ensure rigor in the qualitative phase, the standards recommended by Guba and Lincoln were adopted (37). Before interviews, the first author participated in activities organized by the community senior service center, establishing friendly relationships and gaining participants’ trust. All transcripts and researcher notes were analyzed by two investigators, with regular team discussions throughout the study to enhance the reliability and confirmability of findings. The Creswell and Plano Clark methodology was adopted to identify issues of design quality and interpretive rigor, thereby mitigating potential threats to drawing valid inferences from integrated data (24). Research questions across qualitative and quantitative phases were aligned with the final purpose of synthesizing data. The transferability and credibility of meta-inferences stem from the integration of findings rather than from individual datasets.

### 2.8 Ethics considerations

The study was approved by the Medical Ethics Committee of Jiangsu University (Approval number 2021–719). All methods were conducted in accordance with the Declaration of Helsinki. Written informed consent was obtained from every participant. The research objectives, voluntary participation, confidentiality, and research approval were all documented in the informed consent form. Before data collection, researchers explained the study to participants, including the research objectives, data collection methods, benefits of participation, and the right to withdraw from the study at any time.

## 3 Results

### 3.1 Quantitative survey

#### 3.1.1 Participant characteristics

A total of 445 community-dwelling older adults were recruited, including 212 men (47.6%) and 233 women (52.4%) (Table 1).

**Table 1:** Characteristics of participants in quantitative survey (*N* = 445).

Variables	*N*/%	*p*
Gender		0.006
Male	212 (47.6)	
Female	233 (52.4)	
Age (year)		0.286
60 ~ 74	292 (65.6)	
75 ~ 89	145 (32.6)	
≥90	8 (1.8)	
Residential district		<0.001
Rural	105 (23.6)	
Urban	340 (76.4)	
Education		<0.001
Primary school and below	104 (23.4)	
Junior high school	132 (29.7)	
High school	112 (25.2)	
University and above	97 (21.8)	
Income (RMB, monthly)		<0.001
<1,000	10 (2.2)	
1,000–3,000	93 (20.9)	
3,000–5,000	210 (47.2)	
>5,000	132 (29.7)	
Residence		0.027
Living with a spouse	279 (62.7)	
Living with their children	131 (29.4)	
Living alone	35 (7.9)	
Wear denture		0.711
Yes	210 (47.2)	
No	235 (52.8)	
Oral odor		<0.001
Yes	31 (7.0)	
No	414 (93.0)	
Time since last dental visit		<0.001
<1 year	155 (34.8)	
12–23 months	81 (18.2)	
2–5 years	63 (14.2)	
>5 years or never visiting	146 (32.8)	
Communication with dentists		<0.001
Communication difficulty	54 (12.1)	
No difficulty in communication	391 (87.9)	
Follow-up appointments on schedule		<0.001
Yes	236 (53.0)	
No	209 (47.0)	
Dental floss		<0.001
No	308 (69.2)	
Yes	137 (30.8)	
Brush teeth 2 times		<0.001
No	285 (64.0)	
Yes	160 (36.0)	
Dental cleaning		<0.001
No	264 (59.3)	
Yes	181 (40.7)	

#### 3.1.2 Analysis of factors associated with OHL

The mean OHL score was 10.39 ± 2.99. The mean scores for oral health knowledge, beliefs, and behaviors were 12.40 ± 3.06, 11.38 ± 2.45, and 32.79 ± 6.80, respectively. The mean score for self-efficacy was 77.95 ± 12.37. OHL was positively correlated with oral health behaviors (r = 0.462, *p* < 0.01), as was self-efficacy with oral health behaviors (r = 0.369, *p* < 0.01) (Table 2). Univariate analysis revealed that gender (*p* = 0.006), education (*P <* 0.001), income (*P <* 0.001), residential district(*P <* 0.001), residence (*p* = 0.027), oral odor (*p* < 0.001), communication with dentists (*p* < 0.001), time since last dental visit (*p* < 0.001), follow-up appointments on schedule (*p* < 0.001), dental floss (*p* < 0.001), brush teeth 2 times (*p* < 0.001), and dental cleaning (*p* < 0.001) were significantly associated with OHL among community-dwelling older adults (Table 1). As shown in Table 3, multiple linear regression analysis revealed that oral health knowledge (*β* = 0.074, *p* = 0.032), oral health beliefs (*β* = 0.185, *p* < 0.001), oral health behaviors (*β* = 0.126, *p* = 0.001), not visiting a dentist >5 years or never visiting (*β* = −0.084, *p* = 0.009), difficulty in communicating with the dentist (*β* = −0.075, *p* = 0.010), having a dental cleaning (*β* = 0.297, *p* < 0.001), high school education (*β* = 0.404, *p* < 0.001) or university education (*β* = 0.438, *p* < 0.001), and self-efficacy (*β* = −0.103, *p* = 0.021) were significantly related to OHL levels. The model was statistically significant (*R*^2^ = 0.736, adjusted *R*^2^ = 0.722, *F* = 53.410, *p* < 0.001), explaining a total of 72.2% of the variance in the dependent variable.

**Table 2:** The correlational relationships among variables (*N* = 445).

Variables	Oral health knowledge	Oral health belief	Oral health behavior	Self-efficacy	Oral health literacy
Oral health knowledge	1				
Oral health belief	0.550^*^	1			
Oral health behavior	0.219^*^	0.343^*^	1		
Self-efficacy	0.154^*^	0.279^*^	0.696^*^	1	
Oral health Literacy	0.476^*^	0.548^*^	0.462^*^	0.369^*^	1

*p < 0.01.

**Table 3:** Multivariate regression.

Variables	B (95% CI)	*SE*	*β*	t	*P*	VIF^*^
Constant	6.168 (4.382, 7.955)	0.909		6.788	<0.001	
Oral health knowledge	0.185 (0.016, 0.354)	0.086	0.074	2.155	0.032	1.888
Oral health beliefs	0.317 (0.201, 0.433)	0.059	0.185	5.358	<0.001	1.894
Oral health behavior	0.056 (0.023, 0.089)	0.017	0.126	3.302	0.001	2.312
not visiting a dentist >5 years & never visiting	−0.554 (−0.969, −0.140)	0.211	−0.084	−2.629	0.009	1.635
Difficulty in communicating with dentists	−0.708 (−1.247, −0.169)	0.274	−0.075	−2.580	0.010	1.338
Have a dental cleaning	1.870 (1.299, 2.442)	0.291	0.297	6.438	<0.001	3.398
Self-efficacy	−0.025 (−0.047, −0.004)	0.011	−0.103	−2.309	0.021	3.155
High school education	2.882 (2.358, 3.405)	0.266	0.404	10.824	<0.001	2.227
University education	3.280 (2.664, 3.895)	0.313	0.438	10.472	<0.001	2.790

^*^VIF, Variance Inflation Factor.

### 3.2 Qualitative findings

#### 3.2.1 Participant characteristics

Twenty community-dwelling older adults participated in semi-structured interviews, including 13 women and 7 men, with a mean age of 74.33 ± 6.32 years (Table 4).

**Table 4:** Characteristics of participants in qualitative interview (*N* = 20).

Number	Gender	Age (year)	Marital status	Living with children	Education	Dentures	Number of missing teeth
P1	Female	89	Widowed	No	Primary school	No	30
P2	Male	70	Widowed	Yes	Junior high school	Yes	18
P3	Male	78	Married	No	Primary school	Yes	12
P4	Female	78	Widowed	No	Junior high school	Yes	30
P5	Female	75	Married	Yes	Primary school	No	1
P6	Male	63	Married	Yes	High school	No	1
P7	Female	61	Married	Yes	High school	Yes	1
P8	Female	76	Married	No	Junior high school	No	14
P9	Female	82	Married	No	Primary school	Yes	20
P10	Male	66	Married	Yes	High school	Yes	4
P11	Female	82	Married	No	High school	No	2
P12	Female	72	Married	Yes	University	Yes	1
P13	Male	79	Married	No	Junior high school	Yes	17
P14	Female	84	Married	No	High school	Yes	7
P15	Female	75	Married	No	Junior high school	No	22
P16	Male	77	Married	Yes	University	Yes	4
P17	Female	73	Married	No	High school	Yes	3
P18	Female	72	Married	Yes	Junior high school	No	0
P19	Male	75	Married	No	High school	Yes	2
P20	Female	70	Married	No	Junior high school	No	18

#### 3.2.2 Qualitative interview results

Four themes were identified from interviews explaining the barriers faced by community seniors in accessing, understanding, and processing oral health information (Table 5).

**Table 5:** Qualitative themes describing OHL of community-dwelling older adults.

Theme	subtheme	Description	Participant excerpts
Common misconceptions about oral health	Diminished sensitivity to demand	Most community seniors had a low awareness of their oral health demands. This is primarily reflected in weak oral health awareness and a lack of awareness regarding the necessity of dental examinations and oral hygiene care.	When it comes to health, my view is that as long as (my teeth) can eat, that’s good enough. (P1, female, 89 years) Some individuals are particular about brushing three times a day, but I say that’s just being overly fussy—once a day is plenty. I’ve never understood the need for a second brushing, from childhood to now. (P5, female, 75 years)
		Additionally, some community seniors held negative attitudes such as resignation and acceptance of aging, which further diminished their sensitivity to oral health demands.	The problem with it (the tooth) arises on its own; there’s no way to explain it, and it’s not something people can change. (P10, male, 66 years) Getting on in years, I’m about to kick the bucket (laughs), and going to the dentist just is not worth it anymore, you know. (P8, female, 76 years)
	Insufficient awareness of negative impacts	Community seniors lacked sufficient awareness of the negative impacts of oral diseases. Most community seniors did not clearly understand the adverse effects of oral conditions, failing to give them adequate attention when they arose. This lack of awareness also led community seniors to avoid or delay seeking dentists.	When my gums swell up, I’m the type who does not take medicine—I just tough it out for a few days, and it goes away. (P4, female, 78 years) Sometimes I go (to the dentist), but mostly I do not. When I have a toothache, I just take some painkillers. (P19, male, 75 years)
		Although some older adults recognized the negative impact of oral diseases, their understanding was often limited to the effects on the digestive system.	Poor teeth mean food is not chewed properly, which is also hard on the stomach. (P18, female, 72 years)
Barriers to Accessing Information	Lack of intrinsic motivation	The majority of community seniors lack sufficient intrinsic motivation to seek oral health information and show little initiative in actively obtaining such information.	I do not want to know anything (about oral health). Just like this, every day is fine. As long as I can eat, that’s enough. (P4, female, 78 years)
		Only a few seniors learned about health knowledge through WeChat or television.	I mainly check various WeChat articles (Ah,) and watch doctors giving lectures on a TV show that focuses on health preservation and wellness. I’m learning some medical knowledge. (P20, female, 70 years)
	Incomplete understanding	Low educational attainment limits community seniors’ comprehension of oral health information.	You can still eat without teeth, and you can still go about your life just fine without teeth. (P3, male, 78 years)
		Simultaneously, most community seniors derive their understanding of oral health from elders or personal experience, resulting in a certain degree of bias. This leads to misconceptions about oral health among community seniors.	Toothaches are pretty much universal because, as the old folks say, ‘Toothache is not an illness,’ and I feel that way too. I do not think dental cleanings are really necessary. Some people get them done, and things often go wrong afterward. (P6, male, 63 years)
Significant variations in self-management efficacy		A portion of community seniors demonstrate high self-management efficacy, consistently practicing behaviors such as brushing teeth morning and night, rinsing their mouths, and reducing consumption of sweets.	In the evening, try to eat as little as possible, brush my teeth thoroughly, and avoid eating sweets. (P5, female, 75 years)
		However, some older adults exhibit low self-management efficacy, demonstrating poor self-care habits such as brushing their teeth only once a day or irregularly.	Sometimes I brush my teeth, and sometimes I do not. (P9, female, 82 years) I brush my teeth once in the morning. (P3, male, 78 years)
Lack of trust between patients and dentists		The lack of effective communication between community seniors and dentists has led to a lack of trust in dental professionals among older adults.	Hospitals are all about scamming people out of money. They do not treat illnesses; they just rip people off. (P3, male, 78 years)
		This results in misunderstandings about treatment and unmet healthcare needs among community seniors.	Logically speaking, I’m not satisfied. Because dentures should let me eat and chew properly, right? But these can only chew—they fall out the moment I try to bite down. (P13, male, 79 years)

### 3.3 Data integration

**Figure 2:**
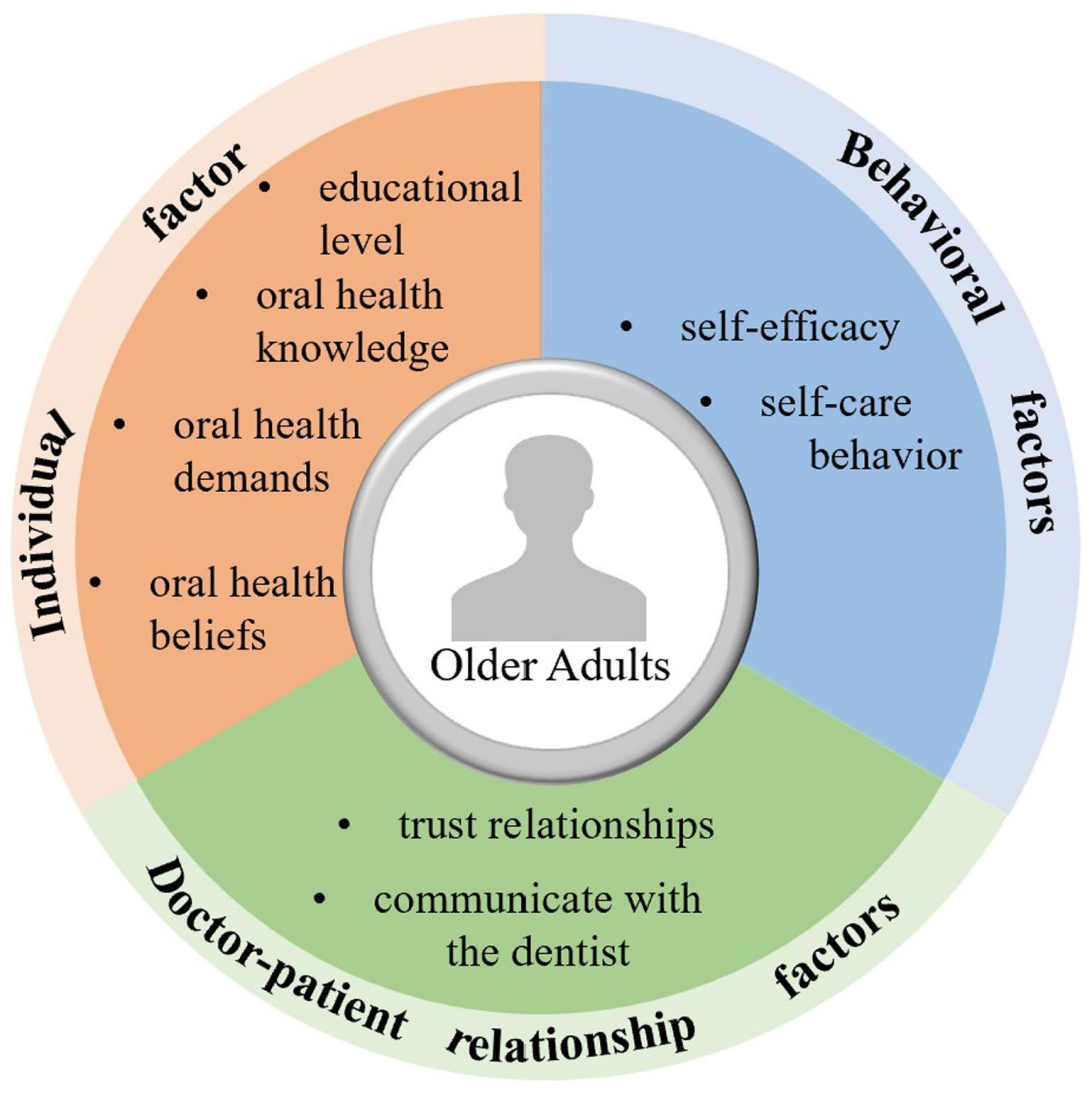
Related factors of OHL among community-dwelling older adults.

We employed a bidirectional approach to integrate data, comparing and merging themes and subthemes from qualitative research with quantitative findings. A research team was established to discuss the final results. The integrated findings are presented in Table 6. Based on the integrated results, we identified the factors associated with OHL among community-dwelling older adults. These included individual factors, behavioral factors, and patient-dentist relationship factors (Figure 2).

**Table 6:** Joint table display of related factors for OHL among community-dwelling older adults.

Themes	Subtheme	Quantitative findings	Meta-inference
Common misconceptions about oral health	Diminished sensitivity to demand	Having a dental cleaning (*β* = 0.297, *p* < 0.001) Oral health behaviors (*β =* 0.126*, p* = 0.001)	Dental cleaning and oral health behaviors were associated with OHL. Qualitative findings further elucidate that perceived oral health demands mediate this relationship. Older adults’ limited awareness of oral health demands and negative attitudes toward healthcare hindered their proactive information-seeking and healthcare-seeking behaviors, thereby lowering their OHL.
	Insufficient awareness of negative impacts	Oral health knowledge (*β =* 0.074, *p* = 0.032)	Oral health knowledge was positively associated with OHL. Qualitative findings illustrate how this association operates: older adults’ knowledge was largely confined to local symptoms of oral diseases and their effects on digestion, with limited awareness of the oral-systemic health connection. This gap weakens knowledge’s contribution to OHL improvement.
Barriers to accessing information	Lack of intrinsic motivation	Oral health belief (*β* = 0.185, *p* < 0.001)	Oral health beliefs were significantly associated with OHL. Qualitative research provides an elaborate explanation. Oral health beliefs influence OHL levels by affecting the internal motivation to seek information. This suggests that information-seeking intent may serve as a key pathway linking oral health beliefs to OHL.
	Incomplete understanding	High school education (*β* = 0.404, *p* < 0.001) or university education (*β* = 0.438, *p* < 0.001) Oral health knowledge (*β* = 0.074, *p* = 0.032)	Higher educational attainment (high school, university, and above) was positively associated with OHL. Qualitative findings extend this by revealing the mechanisms through which education operates: less-educated participants relied on lay sources (older family members or peers, personal experience), resulting in incomplete or biased knowledge, while those with higher education used structured information channels (WeChat, television lectures). Education thus appears to shape OHL partly through information channel quality, beyond cognitive capacity alone.
Variations in self-management efficacy		Self-efficacy (*β* = −0.103, *p* = 0.021) Oral health behaviors (*β =* 0.126*, p* = 0.001) Not visiting a dentist >5 years or never visiting (*β* = −0.084, *p* = 0.009)	Self-efficacy was negatively associated with OHL. Qualitative findings challenge a direct-effect interpretation, showing that higher self-efficacy promotes consistent self-management behaviors (e.g., regular brushing, rinsing). This suggests that self-efficacy may influence OHL indirectly through behavioral pathways rather than directly. The negative regression coefficient may reflect statistical suppression due to strong self-efficacy–behavior collinearity (*r* = 0.696), wherein the behavioral pathway absorbs most of the positive variance attributable to self-efficacy.
Lack of trust between doctors and patients		Difficulty in communicating with dentists (*β* = −0.075, *p* = 0.01)	Difficulties in communicating with dentists were associated with lower OHL. Qualitative data further elucidate the underlying mechanism: poor communication hinders information exchange, leading to misunderstandings regarding oral health and unmet healthcare demands. This erosion of communication also undermines trust between patients and dentists, diminishes treatment adherence, and creates a vicious cycle of “poor communication–low trust–low OHL.”

## 4 Discussion

This study investigated OHL levels among community-dwelling older adults and explored factors associated with OHL. In this convergent mixed-methods study, four meta-themes and four meta-subthemes were identified: common misconceptions about oral health (diminished sensitivity to demand, insufficient awareness of negative impacts), barriers to accessing information (lack of intrinsic motivation, understanding is incomplete), significant variations in self-management efficacy, and lack of trust between doctors and patients. These findings provide a comprehensive perspective on the current OHL levels and related factors among community-dwelling older adults.

The findings revealed that OHL levels among community-dwelling older adults were generally moderate, which is consistent with findings from other studies (38). This research indicates that community-dwelling older adults exhibit misconceptions about oral health and face difficulties in accessing and comprehending relevant information. This may be attributable to their personal attitudes and educational background. Previous studies have demonstrated that OHL is closely associated with attitudes toward oral self-care and educational attainment (29, 39). Many studies have shown that oral health beliefs influence oral health behaviors and oral health status (40, 41), a finding corroborated by the present study. The mixed-methods study suggested that older adults’ oral health beliefs influence their intrinsic motivation to seek information, leading to barriers in information acquisition and affecting their OHL levels. Furthermore, we found that community-dwelling older adults’ understanding of oral health was primarily limited to its importance for the digestive system, with limited awareness of its connection with overall systemic health and chronic diseases. This finding aligns with previous research by Shalinie et al. (42). Therefore, healthcare professionals should strengthen primary prevention efforts regarding oral health among community seniors, with a particular focus on the oral-systemic health link, thereby enhancing their awareness and understanding of oral health. Furthermore, future initiatives to improve OHL should fully take into account the cultural and social backgrounds of older adults and develop targeted, personalized improvement plans.

Furthermore, this study found that diminished demand for oral healthcare was associated with OHL levels. This may be attributed to weak oral health awareness and a lack of recognition of the need for dental examinations and oral care. Previous research has indicated that older adults from rural areas lack awareness of the importance of regular dental cleanings and oral care services, exhibiting lower willingness to participate in and adhere to such services (43). This study also confirmed that dental cleanings and oral health behaviors were associated with OHL. This may be because older adults with lower educational attainment may lack adequate understanding of or training in proper oral hygiene practices, leading them to overlook the importance of regular dental check-ups (44, 45).

Correlation analysis showed a positive association between self-efficacy and OHL (*r =* 0.369, *p* < 0.01). However, multiple regression revealed a negative independent effect of self-efficacy (*β* = −0.103, *p* = 0.021). One possible explanation is that this reflects a suppression effect arising from the strong collinearity between self-efficacy and oral health behaviors (*r* = 0.696). If this interpretation holds, when the behavioral pathway is statistically controlled, the direct effect of self-efficacy on OHL might be attenuated or reversed, perhaps because the remaining variance captures prudent self-assessment tendencies among individuals with high self-efficacy (46, 47). Nevertheless, given the cross-sectional nature of this study, this explanation remains speculative. Research indicates that self-efficacy expectations are significantly associated with both the initiation and maintenance of health behaviors (48). Qualitative findings were consistent with this interpretation: older adults with higher self-efficacy maintained better oral health self-management practices (e.g., brushing twice daily, rinsing after meals, reducing sugar intake), whereas those with low self-efficacy exhibited irregular or inadequate habits. These results tentatively suggest that self-efficacy might exert a positive indirect effect on OHL primarily by promoting oral health behaviors, rather than through a direct positive association. Future studies are needed to confirm this mediation pathway. Consistent with this tentative interpretation, Yang et al. found that self-efficacy served as a mediator between OHL and oral frailty (49). Pending confirmation of this pathway, healthcare professionals might consider addressing older adults’ perceived oral health needs and self-efficacy when developing tailored oral health promotion strategies.

A study indicated that if individuals do not receive health information that is personally meaningful and practically supportive in an appropriate manner, opportunities for meaningful clinical engagement are missed (50). Parnell et al. suggested that health literacy levels depend largely on communication between individuals and healthcare providers (51). Effective communication between dentists and patients is associated with higher OHL, which in turn influences patients’ healthcare decisions (52). Effective communication strategies require a thorough understanding of the target audience, and tailored clinical communication is essential for promoting active patient participation in healthcare activities (53). This study indicates that community-dwelling older adults who lack effective communication with dental professionals exhibit lower OHL. This issue may stem from the fact that community seniors with lower educational attainment struggle to comprehend relevant medical terminology. Consequently, the relationship between these seniors and their dental practitioners has become increasingly distant, even marked by a complete lack of trust. In our qualitative research, some community seniors also mentioned that their healthcare needs were not met or that their misconceptions about oral health care were reinforced, ultimately leading to low OHL. A nationwide survey on dentists’ communication skills revealed that dentists routinely underutilize communication techniques, particularly those considered effective for patients with lower educational attainment (54). Therefore, healthcare providers should further develop their communication skills, maintain patience, and adopt approaches that are more accessible and understandable to older adults, thereby reducing knowledge asymmetry in patient-dentist interactions.

To our knowledge, this study is the first to use a convergent mixed-methods design to investigate OHL and its relevant factors among community-dwelling older adults. This study offers new perspectives for future research in this field. However, this study has some potential limitations. First, quantitative data were collected exclusively in one city, a national pilot city for community-based senior care reform, and participants were recruited from a single community senior service center. OHL levels and service access reported here may be overestimated relative to the broader Chinese older adult population, particularly in under-resourced regions. Caution is therefore needed when generalizing these findings beyond similar pilot settings. Second, all data were self-reported, which may introduce recall or social desirability bias, particularly for sensitive items such as oral hygiene practices and dentist communication experiences. Third, the exclusion criteria (inability to communicate in Mandarin or to perform activities of daily living independently) likely excluded the frailest older adults, including those with cognitive impairment or severe physical disability. Consequently, the findings may not generalize to institutionalized, cognitively impaired, or non-Mandarin-speaking older populations. Furthermore, OHL is susceptible to influences from factors such as geographic location, socioeconomic status, and income level. Future research should comprehensively consider these factors and conduct multi-regional investigations using stratified random sampling.

## 5 Conclusion

This study employed a convergent mixed-methods approach to investigate OHL and its associated factors among community-dwelling older adults. Findings revealed moderate OHL levels linked to multiple factors, including oral health demands, behaviors, knowledge, beliefs, educational level, self-efficacy, and patient-dentist relationships. These findings provide valuable insights for developing intervention strategies to enhance OHL among community-dwelling older adults. Future interventions should focus on improving oral health knowledge, beliefs, behaviors, and self-efficacy.

## Data Availability

The raw data supporting the conclusions of this article will be made available by the authors, without undue reservation.
