# Supplementary material for: Oral health literacy and its related factors among community-dwelling older adults: a mixed-methods study
Source: Front Public Health. 2026 Jul 3;14:1833803. doi: 10.3389/fpubh.2026.1833803 (PMC13376257; doi:10.3389/fpubh.2026.1833803)
Supplement: Supplementary file 1 [file Data_Sheet_1.DOCX]

**Interview guide**

(1) How would you assess your oral health? What role do you think oral health plays in maintaining overall physical health?

(2) How do you acquire information about oral health? And how do you determine whether the information you receive is accurate?

(3) What are your daily oral health habits?

(4) What do you do when you experience oral discomfort? What type of healthcare facility do you typically choose for treating oral diseases? Why?

(5) Do you get a dental checkup or teeth cleaning every year? Why?

(6) When you have questions about oral health, to whom do you turn for help?

**Table 1 Encoding of variables**

| **Variables** | **Encoding** |
| --- | --- |
| Gender | male=1, female=0 |
| Residential District | rural=1, urban=0 |
| Oral odor | yes=1, no=0 |
| Communication with Dentists | yes=1, no=0 |
| Follow-up appointments on schedule | yes=1, no=0 |
| Dental floss | yes=1, no=0 |
| dental cleaning | yes=1, no=0 |
| Brush teeth 2 times | yes=1, no=0 |
| Education | Primary school and below (0,0,0), Junior high school (1,0,0), High school (0,1,0), Above university (0,0,1) |
| Income | ≤5000=0, ＞5000=1 |
| Residence | Living with a spouse or alone=0, Living with their children=1 |
| Distance time to dental appointments | ＜1 year (0,0,0), 12-23 months (1,0,0), 2-5years (0,1,0),＞5 years or never visit (0,0,1) |
